# Potentially inappropriate medications with older people in intensive care and associated factors: a historic cohort study

**DOI:** 10.1590/1516-3180.2022.0666.R1.190523

**Published:** 2023-07-31

**Authors:** Karina Sichieri, Danilo Donizetti Trevisan, Ricardo Luís Barbosa, Silvia Regina Secoli

**Affiliations:** I Nurse and Doctoral Student, Hospital Universitário (HU), Universidade de São Paulo (USP), São Paulo (SP), Brazil.; II PhD. Nurse and Assistant Professor, Universidade Federal de São João Del Rei (UFSJ), Divinópolis (MG), Brazil.; III PhD. Mathematics and Assistant Professor, Universidade Federal de Uberlândia (UFU), Monte Carmelo (MG), Brazil.; IV PhD. Nurse and Senior Professor, Graduate Program in Adult Health Nursing, School of Nursing, Universidade de São Paulo (USP), São Paulo (SP), Brazil.

**Keywords:** Aged, Inappropriate prescribing, Intensive care units, Potentially inappropriate medication list, Potentially inappropriate medications, Older adults, Beers criteria, ICU intensive care units

## Abstract

**BACKGROUND:**

The epidemiology of potentially inappropriate medications (PIMs) in critical care units remains limited, especially in terms of the factors associated with their use.

**OBJECTIVE:**

To estimate the incidence and factors associated with PIMs use in intensive care units.

**DESIGN AND SETTING:**

Historical cohort study was conducted in a high-complexity hospital in Brazil.

**METHODS:**

A retrospective chart review was conducted on 314 patients aged ≥ 60 years who were admitted to intensive care units (ICUs) at a high-complexity hospital in Brazil. The dates were extracted from a “Patient Safety Project” database. A Chi-square test, Student’s t-test, and multivariable logistic regression analyses were performed to assess which factors were associated with PIMs. The statistical significance was set at 5%.

**RESULTS:**

According to Beers’ criteria, 12.8% of the identified drugs were considered inappropriate for the elderly population. The incidence rate of PIMs use was 45.8%. The most frequently used PIMs were metoclopramide, insulin, antipsychotics, non-steroidal anti-inflammatory drugs, and benzodiazepines. Factors associated with PIMs use were the number of medications (odds ratio [OR] = 1.17), length of hospital stay (OR = 1.07), and excessive potential drug interactions (OR = 2.43).

**CONCLUSIONS:**

Approximately half of the older adults in ICUs received PIM. Patients taking PIMs had a longer length of stay in the ICU, higher numbers of medications, and higher numbers of potential drug interactions. In ICUs, the use of explicit methods combined with clinical judgment can contribute to the safety and quality of medication prescriptions.

## INTRODUCTION

Potentially inappropriate medications (PIMs) are those whose potential risks outweigh their benefits for older people when safer alternative therapeutic options exist.^1^ The PIMs use is currently a public health problem worldwide; its magnitude is reflected in epidemiological indicators and negative multidimensional impacts. Rates of PIM use range from 34.6% to 95.8%, depending on the design and other methodological aspects of the studies.^2–6^ Use of potentially inappropriate medications is an important predictor of negative outcomes such as falls, deterioration of patients’ clinical condition, worsening of pre-existing diseases, higher use of health services, and death.^6,7^ In addition to health problems, evidence indicates that using PIM is associated with increased health costs compared to older people who did not use any PIM.^8^

There are two methods of assessing the adequacy of drug prescriptions: explicit and implicit. The implicit method is based on a professional’s clinical judgment, considering the clinical particularities of older people; thus, it is more complex and cannot be reproduced or generalized. At the same time, the explicit method is direct, based on criteria that are usually elaborated upon through expert consensus based on the literature review.^9^ Criteria from different countries are used to classify and describe the risks of PIM use,^10,11^ so part of these criteria are based on the Beers criterion and local differences regarding the drugs approved for use.

The Beers criteria have been used for over 30 years and are the oldest, even as a risk-management tool. A systematic review identified 36 criteria, with lists of drug classes, drug-disease interactions, and drug-drug interactions, considered educational tools that should be included in the comprehensive assessment of all elderly patients who need medication.^12^ Adopting explicit criteria as a way of reviewing the PIM, whether on patient admission or during hospitalization, through computerized systems or not, can help the safe practice of drug prescription for the elderly and reduce the use of PIM in the hospital environment through collaborative work among professionals.^13^ The Beers and other criteria should not replace clinical judgment but serve as a guide for the healthcare team in the daily review of medications to minimize the use of PIMs, and to ensure safe and effective pharmacotherapy in the elderly population.^14^ Updates over the years have made it possible to extend the use of these criteria to contexts involving greater complexity, including units that care for seriously ill patients.^4^

The epidemiology of PIMs in critical care units remains limited, especially in terms of the factors associated with their use. Studies conducted in emergency departments and intensive care units have shown that the predictors include polypharmacy, number of medications prescribed before admission to the intensive care units (ICUs), length of stay, and comorbidities.^7,15–17^ However, studies are inconclusive as to approaches to implementing “inappropriate” criteria in cases where older people are seriously ill. The worsening and severity of chronic and degenerative morbidities, high prevalence of clinical conditions such as pain, anxiety, delirium, age heterogeneity, and complexity of treatment regimens indicate that PIM use may sometimes be appropriate rather than universally inappropriate.

## OBJECTIVE

The present study was designed to estimate the incidence of and factors associated with the use of potentially inappropriate medications prescribed to older adults in intensive care units.

## METHODS

The design was based on the recommendations of the Strengthening the Reporting of Observational Studies in Epidemiology initiative,^18^ considering the adequate development and structuring of the study as well as data presentation.

### Study setting and sample

A historical cohort study was conducted on older patients hospitalized in eight ICUs (clinical, surgical, and specialty) of the Instituto Central Hospital das Clínicas, Faculdade de Medicina da Universidade de São Paulo (HCFMUSP) in Brazil. It is the largest public quaternary care hospital complex in Brazil and cares for patients from various specialties. The consecutive sample consisted of people aged 60 years or older, hospitalized in ICUs, and who used at least one medication. The patients were monitored until they left the ICUs (discharge or death). Older adults who died within the first 24 h were excluded from the study.

### Data sources and variables

The dates were extracted from a “Patient Safety Project” database.^19^ A retrospective chart review was conducted covering all patients aged ≥ 60 years who were admitted to ICUs of the HCFMUSP between September to December 2012. The primary sources of information were patient medical records, which were analyzed by nurses and pharmacists.

A questionnaire was used that included data related to demographic and clinical characteristics, which were: gender (male or female); age (years); type of ICU (clinical; surgical or specialties); type of hospitalization (clinical or surgical) length of stay – length of stay in the ICU (days); mechanical ventilation (Yes or No); renal replacement therapy (Yes or No); nasogastric tubes (Yes or No); condition at the time of discharge from the ICU (survivor or death); Simplified Acute Physiology Score (SAPS) II;^20^ Charlson Scale;^21^ Logistic Organ Dysfunction System (LODS);^22^ and the drug therapy regimens (medications, routes of medication, polypharmacy and drug interactions). Excessive polypharmacy was defined as the daily use of 10 or more medications.^23^ Potential drug-drug interaction (PDDI) analysis was performed for the drug pairs. PDDIs were identified using the Drug Interaction Checker system.^24^

The SAPS II,^20^ measures the severity on the first day of hospitalization. It comprises physiological and demographic variables (age and type of admission) and variables related to specific diseases (acquired immunodeficiency syndrome, metastatic cancer, and hematological malignancy). The worst value of all variables was collected during the first 24 hours after admission to the ICU, and the sum of these points formed the score. The higher the score, the greater the expected severity.

The Charlson Comorbidity Index (CCI) ^21^ is the most widely used and is considered the gold standard for assessing comorbidities in clinical research. The CCI comprises 19 items corresponding to different comorbid medical conditions. Nineteen conditions and their associated weights combined with age were used to predict mortality. The total CCI score consists of a simple sum of weights, with higher scores indicating greater mortality risk and more severe comorbid conditions.

The LODS^22^ evaluates and quantifies organ dysfunction in patients in the ICU. It uses 12 physiological variables and identifies one to three levels of dysfunction in six organ systems: neurological, cardiovascular, renal, pulmonary, hematological, and hepatic. The higher the score, the higher the severity level.

### Identification of PIM use

The dependent variable was the regular use of at least one PIM according to the 2019 version of the Beers criteria,^25^ regardless of the diagnosis and clinical condition. The 2019 version is more appropriate for use in ICUs because it includes drug interactions. This version contains a list of medications and therapeutic classes that older adults should avoid. Medications are divided into several categories based on kidney function: inappropriate medications and classes of medications that should be avoided for older people; medications and classes of medications that should be avoided for older people who have certain syndromes or diseases; medications that should be used with caution; medications that have PDDI that should be avoided for older people; and medications that should be avoided or have their doses adjusted. PIMs were analyzed in relation to the quality of evidence (high, moderate, or low) and strength of evidence (strong, weak, or insufficient).^25^

### Statistical analysis

Statistical analyses were performed using SPSS software (version 20.0, SPSS Inc., Chicago, Illinois, United States, Release 22.0, 2012). The distribution of continuous variables was evaluated using the Shapiro-Wilk test, and the data showed a normal distribution. The chi-square test was used for qualitative variables. Student’s t-test and analysis of variance were used to analyze the quantitative variables. The clinical and therapeutic predictors of PIM use were examined using a multiple logistic regression model. The Hosmer-Lemeshow test was used to assess the fit of the model. Independent variables (P ≤ 0.20 in the bivariate analysis were included in the multiple logistic regression model using a stepwise backward procedure. The criterion for the inclusion of variables was a P value lower than 0.20 in univariate analyses. To obtain the final model, the adjusted coefficients were presented with 95% confidence intervals. The statistical significance was set at 5%.

### Ethical aspects

This study was approved by the local Committee for Ethics in Research (No. 0196/11; June 27, 2011). The study was conducted in accordance with the principles of the Declaration of Helsinki and Medical Research Involving Human Subjects Act.

## RESULTS

### Profile of the drug therapy regimens of the sample

During the study period, 314 elderly individuals were included for a total of 2,158 days, and 24,938 medications were prescribed. Among these, 218 drugs were identified, particularly those that act on the nervous system (21.2%), infections (19.8%), and the cardiovascular system (16.5%). The electrolyte and glucose solutions at different concentrations accounted for 4.6%. Metamizole (100%), metoclopramide (63.0%), heparin (53.5%), and furosemide (44.5%) were administered. Almost all medications were administered intravenously.

According to the Beers criteria, 12.8% (28/218) of the medications were inappropriate for older adults. In the PIM group, more than half (53.6%) of the medications were administered via the parenteral route (subcutaneously, intravenously, or intermittently [89.2%]).

### Older people exposed to PIMs

Approximately half (45.8%) of the older adults (n = 314) were exposed to PIMs. Among them, 59.6% received two to four PIMs, and 29.8% received more than five PIMs. Table 1 presents the sample profiles. Age group, ICU type, polypharmacy, mechanical ventilation, and potential drug interactions were significantly associated (P < 0.05).

**Table 1:** Demographic and clinical characteristics of the sample

Demographic variable and clinical evolution		No PIM n = 170 n (%)	Yes PIM n = 144 n (%)	Total	P*
**Gender**					0.179
	Male	84 (49.1)	83 (57.6)	167 (53.2)	
	Female	86 (50.9)	61 (42.4)	147 (46.8)	
**Age**					**0.045**
	60 to 74 years old	106 (62.3)	106 (73.6)	212 (67.5)	
	75 or older	64 (37.7)	38 (26.9)	102 (32.5)	
**Type of ICU**					**0.011**
	Surgery (general)	77 (45.3)	42 (29.2)	119 (37.9)	
	Clinical (internal medicine and pulmonology)	58 (34.1)	59 (40.9)	117 (37.2)	
	Specialties (burns, neurology, nephrology and infectious diseases)	35 (20.6)	43 (29.9)	78 (24.9)	
**Type of hospitalization**					0.536
	Clinical	106 (62.3)	98 (68.1)	204 (65)	
	Elective surgery	34 (20)	26 (18.1)	60 (19.1)	
	Emergency surgery	30 (17.7)	20 (13.8)	50 (15.9)	
**Polypharmacy**					< **0.001**
	Yes	95 (55.9)	117 (81.2)	212 (67.5)	
	No	75 (44.1)	27 (18.8)	102 (32.5)	
**Drug interactions** **					< **0.001**
	Up to 10 pairs	98 (59.4)	31 (21.5)	129 (41.7)	
	≥ 10 pairs	67 (40.6)	113 (78.5)	180 (58.3)	
**Mechanical ventilation**					**0.036**
	Yes	65 (38.4)	73 (50.7)	138 (43.9)	
	No	105 (61.6)	71 (49.3)	176 (56.1)	
**Renal replacement therapy**					0.158
	Yes	21 (12.3)	27 (18.7)	48 (15.3)	
	No	149 (87.7)	117 (81.3)	266 (84.7)	
**Nasoenteral tubes**					0.719
	Yes	57 (33.5)	52 (36.1)	109 (34.7)	
	No	113 (66.4)	92 (63.9)	205 (65.3)	
**Death**					0.074
	Yes	132 (77.6)	98 (68.1)	84 (26.8)	
	No	38 (22.4)	46 (31.9)	230 (73.2)	

PIM = potentially inappropriate medications; ICU = intensive care unit.

* P value obtained through Chi squire test;

** n = 309 older people who had potential drug-drug interactions.

Patients taking PIMs had longer lengths of stay in the ICU (P = 0.008), higher numbers of medications (P < 0.001), and higher numbers of PDDIs (P < 0.001), as illustrated in Table 2.

**Table 2:** Statistics analysis for severity, clinical evolution and therapy indicators in the intensive care unit

Variables	No PIMs	Yes PIMs	P*
	n = 170	n = 144	
	Mean (SD)	Mean (SD)	
Length of stay	4.26 (4.58)	8.15 (9.14)	**0.001**
SAPS II (%)	0.20 (0.22)	0.17 (0.18)	0.834
LODS (%)	0.28 (0.24)	0.27 (0.23)	0.555
Charlson	1.87 (1.91)	1.72 (1.87)	0.428
No. of medications	10.34 (3.27)	12.99 (3.39)	**0.001**
Total no. of PDDIs	12.91 (13.45)	29.52 (26.89)	**0.001**

PIM = potentially inappropriate medications; SD = standard deviation; SAPS II = simplified acute physiology score; LODS = logistic organ dysfunction system; PDDI = potential drug-drug interaction.

* P value obtained through analysis of variance.

For all PIMs, the recommendations to avoid use were classified as strong, and half (50%) of the quality of evidence was classified as high. The most frequently used PIMs were metoclopramide, insulin, antipsychotics, nonsteroidal anti-inflammatories, and benzodiazepines (Table 3).

**Table 3:** Potentially inappropriate medications and rationale, quality of evidence, and strength of recommendation

Organ system, therapeutic category, drug	Patients n (%)	Rationale	Quality of evidence	Strength of recommendation
Metoclopramide	138 (95.8)	Can cause extrapyramidal effects, including tardive dyskinesia; risk may be greater in frail older adults.	Moderate	Strong
Insulin	133 (92.4)	Higher risk of hypoglycemia without improvement in hyperglycemia management, regardless of care setting	Moderate	Strong
Antipsychotics Chlorpromazine Haloperidol Quetiapine Olanzapine	96 (66.7)	Increased risk of cerebrovascular accident (stroke) and higher rate of cognitive decline and mortality in persons with dementia.	Moderate	Strong
NSAIDs Aspirin Ketoprofen Naproxen Ibuprofen	78 (54.1)	Increased risk of gastrointestinal bleeding or peptic ulcer disease in high-risk groups, including those aged > 75 or taking oral or parenteral corticosteroids, anticoagulants, or antiplatelet agents.	Moderate	Strong
BZD Clonazepam Diazepam Lorazepam	75 (52.1)	Older adults have increased sensitivity to benzodiazepines and decreased metabolism of long-acting agents.	Moderate	Strong
Amiodarone	47 (32.6)	Effective for maintaining sinus rhythm but has greater toxicity than other antiarrhythmics used in atrial fibrillation	High	Strong
TCA alone or in combination Amitriptyline Nortriptyline Paroxetine Sertraline	27 (18.7)	Highly anticholinergic, sedating and causes orthostatic hypotension.	High	Strong
Mineral oil, given orally	18 (12.5)	Potential for aspiration and adverse effects; safer alternatives available.	Moderate	Strong
First-generation antihistamines Diphenhydramine Hydroxyzine Promethazine	13 (9)	Highly anticholinergic; clearance reduced with advanced age, and tolerance develops when used as hypnotic; risk of confusion, dry mouth, constipation, and other anticholinergic effects or toxicity	Moderate	Strong
Antispasmodics Atropine Scopolamine	12 (8.4)	Highly anticholinergic, uncertain effectiveness.	Moderate	Strong
Hypnotics Zolpidem	8 (5.5)	Adverse events similar to those of benzodiazepines in older adults (e.g., delirium, falls, fractures);	Moderate	Strong
Digoxin	6 (4.2)	Should not be used as a first-line agent in atrial fibrillation, because there are safer and more effective alternatives for rate control supported by high-quality evidence	Low/ Moderate	Strong
Phenobarbital	5 (3.5)	High rate of physical dependence, tolerance to sleep benefits, greater risk of overdose at low dosages	High	Strong

NSAIDs = nonsteroidal anti-inflammatory drugs; BDZ = benzodiazepines; TCA = tricyclic antidepressants.

Analysis of the patients for whom PIMs were most frequently prescribed showed that among older people who needed mechanical ventilation (n = 73), over a third (38.5%) received antipsychotics, and almost half (46.5%) received at least one benzodiazepine. In the group of older adults with nasoenteral tubes (n = 52), half received antipsychotics and 44.2% received benzodiazepines. Metoclopramide was prescribed to most catheter users (80.7%) and older people on mechanical ventilation (89%).

Drugs that act on the central nervous system are often involved in drug-drug interactions. Analysis of drug pairs showed that the most frequently prescribed combinations were haloperidol + opioid analgesics (morphine, tramadol, methadone, and fentanyl (40.3%), midazolam + fentanyl (36.1%), and fentanyl or morphine + tramadol (33.4%]) (Table 4). Factors associated with PIM use were the length of stay (odds ratio [OR] = 1.07), number of medications (OR = 1.17), and potential drug-drug interactions (OR = 2.43) (Table 5).

**Table 4:** Drug interactions that should be avoided in older people and risk rationales

Object drug and class	Interacting drug and class	Risk rationale	Patients n (%)
Anticholinergics	Anticholinergics	Increased risk of cognitive decline	32 (22.2)
Antidepressants (i.e., TCAs^b^ and SSRIs^c^)	≥ 2 other CNS-active drugs^a^	Increased risk of falls	35 (24.3)
Antipsychotics	≥ 2 other CNS-active drugs^a^	Increased risk of falls	96 (66.7)
Benzodiazepines and nonbenzodiazepines, benzodiazepine receptor agonist hypnotics	≥ 2 other CNS-active drugs^a^	Increased risk of falls and fractures	62 (43.1)
Opioid receptor agonist analgesics	≥ 2 other CNS-active drugs^a^	Increased risk of falls	50 (34.7)
Warfarin	Amiodarone	Increase risk of bleeding	24 (16.7)

TCA = tricyclic antidepressants; SSRIs = selective serotonin reuptake inhibitors; CNS = central nervous system.

a CNS-active drugs: antipsychotics, benzodiazepines, non-benzodiazepines, benzodiazepine receptors against hypnotics,

b tricyclic antidepressants,

c selective serotonin reuptake inhibitors, and opioids.

**Table 5:** Logistic regression analysis of use of potentially inappropriate medications

Variables	OR	CI 95%	P*
Length of stay	1.074	1.020–1.131	0.007
Number. of drugs	1.173	1.059–1.299	0.002
PDDIs ≥ 10 pairs	2.430	1.189–4.965	0.015

OR = odds ratio; CI = confidence interval, PDDIs = potential drug-drug interactions.

* P value obtained using the multiple logistic regression model.

## DISCUSSION

Despite the widespread dissemination of information regarding the effects of medications on older people and the potential negative outcomes associated with PIM use, drug therapy regimens are still being used worldwide that could compromise the quality of care, exposing older people to significant safety risks. In the present study, approximately half of the older adults received at least one PIM on an ongoing basis. This finding is supported by previous studies showing that PIMs are widely prescribed in critical care settings, although there are alternative medications available in the markets of various countries.^6,7,15,26^

Older adults often have chronic diseases that worsen throughout their lives and require hospitalization in critical care units. The present study found that most patients (70.8 %) who received PIMs were in clinical or specialty ICUs (burns, infectious diseases, nephrology, and pulmonology). Therefore, PIMs may have been administered before ICU admission. Studies have shown that 20.6%–68.3% of older people admitted to ICUs received PIMs before admission because of preexisting diseases.^7,26–29^ The PIMs that used the most - metoclopramide, insulin, and antipsychotics, including drug interactions, were medications and/or combinations often used as therapeutic strategies. Thus, some PIMs may have been appropriately prescribed. There are several possible explanations for these results.

The widespread prescription of metoclopramide may have been influenced by several factors, including its low cost and the need for increased motility of the gastrointestinal tract as a result of the prolonged use of parenteral nutrition or paralytic ileus, especially in critically ill older people. This dopaminergic antagonist, with antiemetic and gastroprokinetic properties, may have been used to improve the success rate of post-pyloric placement of nasojejunal tubes and increase patient tolerance to enteral nutrition. More than one-third (36.1%) of patients who received PIMs were on catheters. Metoclopramide may also be a simple preventive strategy. When catheters are inserted, this agent reduces the risk of aspiration and the incidence of pneumonia.^30,31^

Despite the risk of hypoglycemia without improving hyperglycemia management,^25^ insulin was prescribed to almost all patients. A possible explanation for this is the use of glycemic control protocols. Hyperglycemia frequently occurs in critically ill patients and is considered potentially toxic, as it increases the risk of inflammatory and thrombotic events that can contribute to the occurrence of multiple organ and system dysfunction and mortality.^32^ The potential benefits of insulin infusion for glycemic control have been demonstrated in studies conducted with critically ill patients, including older people, which have undoubtedly contributed to the prescription of this PIM.^33,34^

Explicit criteria such as the Beers criteria facilitate PIM reviews during a patient’s hospital journey. They can be recommended to prescribers with an interprofessional approach or incorporated into the clinical decision to guide appropriate prescription, thus reducing unnecessary PIM use. However, these criteria should not replace the clinical judgment of health professionals regarding the patient’s condition. Knowledge of these tools can help health teams minimize the dosage and/or duration of PIM to avoid potential adverse drug events in the elderly.^14^

In the analysis of drug interactions that should be avoided in older adults, those involving drugs that act on the central nervous system (CNS; opioid analgesics, benzodiazepines, and antipsychotics) are particularly noteworthy. The concomitant use of three or more psychotropic drugs has been verified, a finding corroborated by another study conducted on critically ill patients.^6,7^

Almost the entire sample (86.1%) received at least one combination of an object drug and two or more interacting drugs, which increases the risk of falls.^25^ The evaluation of the risk and benefit ratio of these combined regimens must be contextualized for critical care situations, especially because a significant portion of the sample was subjected to therapy recommended in guidelines. It can be inferred that the pain, agitation, and delirium triad commonly observed in critically ill patients, although not directly measured, may have been frequent in this sample.^35^

Clinical conditions that often overlap require multimodal regimens that include opioid analgesics, benzodiazepines, and other non-benzodiazepine sedatives, which were widely prescribed to older people in this study. A combination of opioid analgesics and benzodiazepines may have been used to enhance the patient’s comfort. Opioid analgesics are the first-line agents for treating non-neuropathic pain in critically ill patients, and when combined with benzodiazepines, they can induce deep sedation.^35^

Despite evidence indicating that propofol and dexmedetomidine are more acceptable therapeutic options for sedation,^36^ benzodiazepines are the main agents used for sedation, which is consistent with previous studies.^37,38^ Moreover, despite the versatile therapeutic actions of benzodiazepines, older people are significantly more prone to sedative effects, especially when there are drug combinations, such as those in this sample.

Most of the older adults (66.7%) received combinations that included antipsychotics. This therapeutic class reduces excessive dopaminergic activity, a major contributing factor in delirium, which favors its prescription in critical care situations.^39,40^ Studies have shown that delirium affects a significant proportion of older people in ICUs and that haloperidol, the predominant antipsychotic in the study sample, prevents delirium.^39,40^

In patients receiving mechanical ventilation, agitation, which often results from delirium, increases the risk of extubation. Among older adults with nasoenteral tubes, confusion, which is also associated with delirium, can lead to accidental tube removal. This explanation is supported by the findings of this study. More than one-third (38.5 %) of the patients receiving PIMs on mechanical ventilation were prescribed antipsychotics. Among the patients with nasoenteral tubes, 44.2% received haloperidol or chlorpromazine.

In this cohort, a higher number of medications, which reflects polypharmacy, longer lengths of stay, and an excessive number of potential drug interactions were associated with PIM use. Because of these relationships, the variables should be interpreted together.

In critical care situations, studies are virtually unanimous in identifying an association between polypharmacy and PIMs,^2,3,5^ which tend to coexist, especially during prolonged hospital stays. The average length of stay (8.15 days) of patients who received PIMs was almost twice that of those who did not receive PIMs (4.26 days). The average number of medications administered to patients who received PIMs was also higher (12.99). Among older adults who remain hospitalized for longer periods, it is expected that there will be more opportunities for drug consumption and more occurrences of PDDIs from therapeutic combinations, as evidenced in the sample.

In teaching hospitals, such as the setting in this study, the longer the length of stay in the ICU, the greater the possibility that different physicians are responsible for prescribing drugs. This aspect plays an important role in PDDIs. The risk of an older person receiving an inappropriate therapeutic combination is directly related to the number of prescribers.^41^

The excessive number of potential drug interaction predictors for PIMs is unprecedented and reflects the use of complex therapeutic regimens. In critical care situations, there is an overlapping need to treat acute chronic diseases; control clinical conditions such as pain, agitation, and delirium; and prevent complications, leading to the use of multimodal protocols. The combinations noted in this study can be used as therapeutic strategies, and many were, in fact, necessary. However, the use of such combinations can also increase the toxicity of the agents, particularly in cases where their anticholinergic effects overlap, as observed in this study. In critical care situations, although the Beers criteria provide excellent guidelines for assessing the quality of prescriptions, this tool must be used in conjunction with clinical judgment and patient-centered care. Owing to the complexity of patient conditions, many PIMs are reasonably appropriate for their clinical condition.

Although it used a convenience sample and had some limitations, the findings of the present study must be interpreted at the epidemiological level. A sample of critically ill older people hospitalized in Brazil´s largest hospital complex, whose clinical staff was responsible for medical education at Latin America’s largest university, was analyzed. Although the present study was retrospective, the drugs prescribed, including PIMs, are still used in current therapeutic regimens, especially in multimodal regimens. The lack of information on the dosage and duration of PIM therapy, therapeutic class duplication, and indications according to clinical conditions may have influenced the estimation of PIM use. Differences in the profile of PIM use in ICUs were not assessed, an aspect that would definitely reveal particularities according to specialty area. Future studies may overcome these limitations and increase the knowledge of the subject.

## CONCLUSION

In summary, the study showed that approximately half (45.8%) of the older adults in the ICUs received a PIM or therapeutic combination that may often be appropriate for the clinical situation. PIM use is associated with longer hospitalization, polypharmacy, and an excessive number of potential drug interactions. These factors, which are predictors of adverse events in older adults, coexist in critical care settings. Within the context of ICUs, the use of explicit methods, such as the Beers Criteria, combined with clinical judgment (implicit criteria) can contribute to the safety and quality of the prescription of medications, despite the challenge of selecting the appropriate treatment due to the heterogeneity of patients.
